# Dietary Choline Intake During Pregnancy and Congenital Heart Defects in a Chinese Population

**DOI:** 10.3390/nu18010126

**Published:** 2025-12-31

**Authors:** Yue-Hua Li, Ziqi Xiao, Rui Guo, Baligen Rekemubieke, Wanting Hu, Xin Liu, Jiaomei Yang

**Affiliations:** 1 Department of Epidemiology and Biostatistics, School of Public Health, Xi’an Jiaotong University Health Science Center, Xi’an 710061, China; 2 Key Laboratory of Environment and Genes Related to Diseases, Xi’an Jiaotong University, Ministry of Education, Xi’an 710061, China

**Keywords:** choline, pregnancy, congenital heart defects, phosphatidylcholine, sphingomyelin, free choline, glycerophosphocholine, phosphocholine

## Abstract

**Background/Objectives**: The impact of choline on congenital heart defects (CHDs) in humans remains unclear. This study aimed to investigate the associations between maternal dietary intakes of choline and choline derivatives during pregnancy and CHD. **Methods**: This case–control study included 474 cases and 948 controls from hospitals in Northwest China. Pregnant women admitted for delivery were enrolled and completed a validated food frequency questionnaire to assess their dietary intake during pregnancy. A standardized questionnaire was also administered to collect additional pregnancy-related information. Mixed logistic regression models were used to estimate ORs (95%CIs) for CHD in association with choline intake. **Results**: Higher intakes of total choline, phosphatidylcholine, sphingomyelin, glycerophosphocholine, and phosphocholine in pregnancy were associated with reduced risks of total CHD, ventricular septal defects, and atrial septal defects, with all trend tests showing statistical significance (all *p* < 0.05). The ORs (95%CIs) of total CHD, comparing the highest with the lowest tertiles of intake, were 0.38 (0.24–0.61) for total choline, 0.51 (0.38–0.70) for phosphatidylcholine, 0.37 (0.26–0.51) for sphingomyelin, 0.34 (0.21–0.53) for glycerophosphocholine, and 0.53 (0.34–0.82) for phosphocholine. The inverse associations remained unchanged according to maternal age, work, education, parity, passive smoking, anemia, medication use, or folate/iron supplements use in pregnancy; however, these associations appeared to be more pronounced among pregnant women in urban areas. **Conclusions**: Higher maternal intake of dietary choline during pregnancy may be associated with a lower risk of CHD. Promoting choline intake in pregnant women could serve as a potential strategy for the primary prevention of fetal CHD in China.

## 1. Introduction

Congenital heart defects (CHD) comprise the most common group of congenital malformations worldwide [1]. CHD affects around 1 in 100 newborns in the world [1], and caused more than 0.26 million deaths in 2017 [2], causing great burdens on families and society [2]. The prevalence of CHD at birth has increased in China during the last few decades [3,4], with over 150,000 incident cases yearly [3]. Previous studies have indicated that both genetic and environmental factors may be associated with the development of the fetal cardiovascular system [5]; however, the etiologies of CHD remain largely unknown [5]. It is warranted to identify modifiable risk factors for CHD to provide evidence for primary prevention.

Maternal nutrition in pregnancy, a primary modifiable factor, is crucial for fetal development [6,7,8]. Previous studies have observed that several nutrients in one-carbon metabolism (a critical network of folate-driven biochemical reactions essential for nucleotide synthesis and methylation), including folate, vitamin B_12_, and zinc, are associated with a lower CHD risk [9,10,11], possibly through the reduction in homocysteine concentration that affects fetal heart development [9,10,11]. Choline, a precursor for membrane phospholipids and the neurotransmitter acetylcholine, is also involved in one-carbon metabolism for the conversion of homocysteine to methionine [12]. One animal study showed that maternal choline deficiency in pregnancy affected cardiac development in mice [13]. However, the association between choline and CHD in humans is still unclear, with limited related studies. Two prior studies with small sample sizes investigated maternal choline concentration in CHD cases compared to normal controls [14,15], yielding conflicting results—one reported a significant difference [14], while another found no significant difference [15]. Another study found some CHD subtypes were associated with low intakes of methyl nutrients, like choline, among American pregnant women [16]. Moreover, to our knowledge, no prior studies have specifically examined the associations between different choline derivatives in pregnancy and CHD, including lipid-soluble choline, such as phosphatidylcholine and sphingomyelin, and water-soluble choline, such as free choline, glycerophosphorylcholine, and phosphorylcholine. Such a knowledge gap warrants addressing to optimize prenatal nutrient supplementation for the primary prevention of fetal CHD.

Therefore, we conducted a case–control study to explore the associations between maternal intakes of total choline and choline derivatives (phosphatidylcholine, sphingomyelin, free choline, glycerophosphocholine, and phosphocholine) during pregnancy and CHD in a Chinese population.

## 2. Materials and Methods

### 2.1. Study Design and Participants

A case–control study was carried out across six hospitals in Xi’an, China, from August 2014 to August 2016. Detailed fetal echocardiography was part of the routine prenatal screening program across the six cooperating hospitals during the 20th to 24th weeks of gestation, serving as a prenatal diagnosis for CHD. Previous studies have reported the study design in detail [10,17,18]. Briefly, pregnant women were recruited when awaiting delivery in the obstetrics departments of the cooperated hospitals. Women whose fetuses had isolated CHD without any gene disorders were included in the cases, while women whose fetuses had no congenital abnormalities, including CHD, neural tube defects, orofacial clefts, or limb defects, were included in the controls. Women with multiple gestations or gestational diabetes were excluded because of potential heterogeneity. Qualified professionals from obstetrics, ultrasound, and pediatrics departments in each hospital strictly and rigorously adhered to the standard diagnostic criteria. A follow-up by telephone was conducted within twelve months after birth to confirm the diagnoses. The controls were selected in each hospital each month, maintaining a 2:1 ratio of controls to cases within the same month and hospital. To detect a significant OR of 0.72 (*p* < 0.05) with 80% statistical power, assuming a 50% exposure prevalence in the control group for a two-group comparison, 886 controls and 443 cases were required. A total of 948 controls and 474 cases were included in the analyses, fulfilling the sample size requirements. The post hoc power analyses using the actual data showed that even with the tertile comparison, the statistical power to detect the actual effect sizes exceeded 99%.

This study was approved by the Xi’an Jiaotong University Health Science Center (No.2012008) on 3 March 2012. All participants provided written informed consents.

### 2.2. Dietary Assessment

Pregnant women who were waiting for delivery during the third trimester of pregnancy in hospitals were interviewed to report their diets during the whole pregnancy by a semi-quantitative food frequency questionnaire (FFQ). The median interval between the interview completion and delivery date was two days in both cases and controls. Maternal dietary habits tend to be stable across pregnancy [19]; thus, maternal diets throughout pregnancy are similar to those in the critical period of cardiac development in the third to eighth gestational weeks [10,17,18]. The FFQ was constructed on the basis of a validated FFQ for pregnant women in Northwest China [20], which included a list of 111 food items commonly consumed by local residents. Participants were asked to report the frequency of the consumed food from eight predefined groups (never, <1 time per month, 1–3 times per month, 1 time per week, 2–4 times per week, 5–6 times per week, 1 time per day, and ≥ times per day). To improve the accuracy of portion size estimation, the questionnaire was supplemented with color images of food portions, which depicted small, medium, and large serving sizes. The intake of nutrients was calculated by multiplying the consumption frequency of each food by the nutrient content in the specified portion size. Due to the lack of available data on choline content in Chinese foods, dietary choline content is based on the food composition from the United States Department of Agriculture (USDA) [21,22], which has been widely applied and validated in nutritional studies involving Chinese and other Western populations [23,24,25]. Total choline is formed from lipid-soluble choline (phosphatidylcholine and sphingomyelin) and water-soluble choline (free choline, glycerophosphocholine, and phosphocholine). Dietary supplements during pregnancy were also collected in the interviews; however, no choline supplements were reported among the study participants.

### 2.3. Covariates

Information on maternal socio-demographic and lifestyle factors in pregnancy was collected using a standard questionnaire. The study covariates included maternal age (≥30 years/<30 years), work (in employment/without employment), education (senior high school or above/junior high school or below), residence (urban/rural), parity (≥1/0), passive smoking (no/yes), anemia (no/yes), medication use (no/yes), folate/iron supplements use (no/yes), and dietary diversity score in pregnancy. Women who engaged in paid work outside the household were classified as in employment. Anemia was diagnosed in pregnant women with a hemoglobin concentration below 110 g/L. The dietary diversity score was constructed using the sum of ten defined food group scores according to the methods previously reported [17].

### 2.4. Statistical Analyses

Baseline data were compared among groups by χ2 test for categorical variables, and Mann–Whitney U test or Kruskal–Wallis test for continuous variables due to their non-normal distribution as determined by the Shapiro–Wilk test. Considering the clustering in the design through hospitals, the mixed logistic regression model was adopted to determine ORs (95%CIs) for total CHD and the common subtypes in association with dietary choline intake (total choline, phosphatidylcholine, sphingomyelin, free choline, glycerophosphocholine, and phosphocholine) in pregnancy. Dietary choline intake was categorized by tertiles of the control distribution. Confounding factors were selected by previous studies [17,18], and the estimated change by more than 10% [26]. Model 1 was adjusted for total energy intake in pregnancy. Model 2 was adjusted for total energy intake in pregnancy and sociodemographic factors (maternal age, work, education, residence, and parity). Model 3 was adjusted for all factors in Model 2 plus maternal health-related factors (passive smoking, anemia, medication use, folate/iron supplements use, and dietary diversity score in pregnancy). These models progressively included confounders representing various aspects closely related to CHD from Model 1 to Model 3, which allowed for an assessment of the robustness of the results. Since the intakes of choline derivatives were highly correlated (Table S1), they were not mutually adjusted to avoid multicollinearity in the models [23]. The trend test was conducted by including tertile-specific median intake in the model. Similar analyses were performed to evaluate the associations between maternal intakes of lipid-soluble choline (phosphatidylcholine and sphingomyelin) and water-soluble choline (free choline, glycerophosphorylcholine, and phosphorylcholine) during pregnancy and CHD. To further explore the shapes of the associations of dietary choline intake with CHD, the restricted cubic spline (RCS) curves with three knots were plotted, as this configuration yielded the lowest Akaike Information Criterion compared to models with four or five knots. The linearity assumption was tested by comparing the model fit between the linear and spline models using a likelihood ratio test. The threshold intake level for each nutrient was defined as the point on the RCS curve beyond which the OR plateaued. There were no missing values on the variables of interest in the analyses.

To explore potential heterogeneity, CHD cases were categorized into simple and severe types according to established clinical classifications [27]. Subgroup analyses were conducted according to baseline characteristics, including maternal age, occupation, education, parity, passive smoking, anemia, medication use, or folate/iron supplement use in pregnancy. The interactions were tested by incorporating cross-product terms in the regression models. Sensitivity analyses were conducted by using the residual energy-adjusted intakes of total choline and choline derivatives as exposures, and additional adjustments for dietary intakes of other one-carbon metabolism nutrients, including folate, vitamin B_12_, betaine, and methionine. Sensitivity analyses were also performed by restricting analyses to mothers taking folate supplements in early pregnancy.

The statistical analyses were performed with Stata software (version 15.0; StataCorp, College Station, TX, USA), and a two-sided *p* < 0.05 was considered statistically significant.

## 3. Results

### 3.1. Characteristics of Participants

Among the 474 fetuses diagnosed with CHD, 222 (46.8%) had ventricular septal defects (VSD), and 218 (46.0%) had atrial septal defects (ASD) (Table S2). Other common observed types of CHD included atrioventricular septal defects, patent ductus arteriosus, and tetralogy of Fallot. In total, 311 (65.6%) cases were categorized as simple CHD, and 163 (34.4%) as severe CHD. Pregnant women in the case group had a lower intake of total choline than those in the control group (all *p* < 0.001), with medians (25th percentile, 75th percentile) of 190.4 (120.1, 256.7) mg/d and 246.9 (188.1, 322.2) mg/d, respectively (Table 1). Differences in dietary intakes of choline derivatives (phosphatidylcholine, sphingomyelin, free choline, glycerophosphocholine, and phosphocholine), sociodemographic characteristics (maternal work, education, residence, and parity), and health-related factors in pregnancy (passive smoking, anemia, medication use, and folate/iron supplements use) existed among cases and controls (all *p* < 0.05). The characteristics of study participants by tertiles of total choline intake are displayed in Table S3. In the case group, participants with a higher intake of total choline intake tended to have work, have a higher educational level, live in a rural area, and be nulliparous (all *p* < 0.05). In the control group, participants with a higher intake of total choline intake tended to have a higher educational level, live in urban areas, and not take folate/iron supplements (all *p* < 0.05). Pregnant women with a higher intake of total choline were more likely to have higher intakes of choline derivatives, including phosphatidylcholine, sphingomyelin, free choline, glycerophosphocholine, and phosphocholine (all *p* < 0.001).

### 3.2. Associations of Choline Intake During Pregnancy with Congenital Heart Defects

The risk for total CHD decreased with increasing intakes of total choline, phosphatidylcholine, sphingomyelin, glycerophosphocholine, and phosphocholine during pregnancy, with all trend tests showing statistical significance (all *p* < 0.05) (Table 2). Compared with those in the lowest tertile of total choline intake, participants in the medium and highest tertiles had a lower risk of total CHD (medium vs. lowest tertile: OR = 0.53, 95%CI = 0.38–0.73; highest vs. lowest tertile: OR = 0.38, 95%CI = 0.24–0.61). For each 50 mg increase in total choline intake in pregnancy, the risk for total CHD was reduced by 16% (OR = 0.84, 95%CI = 0.76–0.92). The ORs (95%CIs) comparing the extreme tertiles of phosphatidylcholine, sphingomyelin, glycerophosphocholine, and phosphocholine were 0.51 (0.38–0.70), 0.37 (0.26–0.51), 0.34 (0.21–0.53), and 0.53 (0.34–0.82), respectively. For every 50 mg increase in maternal intakes of phosphatidylcholine, sphingomyelin, glycerophosphocholine, and phosphocholine during pregnancy, the risk of total CHD decreased by 28% (OR = 0.72, 95%CI = 0.63–0.83), 41% (OR = 0.59, 95%CI = 0.36–0.96), 85% (OR = 0.15, 95%CI = 0.07–0.31), and 84% (OR = 0.16, 95%CI = 0.05–0.49), respectively. Similarly, the risks of VSD and ASD were reduced with increasing intakes of total choline, phosphatidylcholine, sphingomyelin, glycerophosphocholine, and phosphocholine in pregnancy, with all the tests for trends indicating significance (all *p* < 0.05) (Tables S4 and S5). However, no significant associations between free choline intake during pregnancy and either total CHD, VSD, or ASD were observed.

The associations of choline intakes from lipid-soluble and water-soluble sources during pregnancy with total CHD and the subtypes are further present in Table 3 and Table S6. The risks of total CHD, VSD, and ASD were reduced with increasing choline intakes from both lipid-soluble and water-soluble sources in pregnancy, with all tests for trends indicating significance (all *p* < 0.05). The ORs (95%CIs) comparing the extreme tertiles of lipid-soluble and water-soluble choline were 0.40 (0.27–0.61) and 0.51 (0.31–0.84), respectively. For each 50 mg increase in lipid-soluble and water-soluble choline intakes during pregnancy, the risk of total CHD was reduced by 26% (OR = 0.74, 95%CI = 0.65–0.84) and 32% (OR = 0.68, 95%CI = 0.51–0.89), respectively.

**Figure 1:**
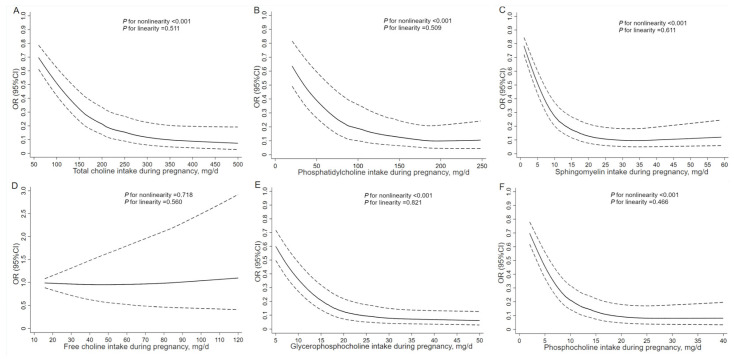
Restricted cubic spline models for the associations of dietary choline intake during pregnancy with total congenital heart defects. Analyses were adjusted for total energy intake, maternal age, work, education, residence, parity, passive smoking, anemia, medication use, folate/iron supplements use, and dietary diversity score in pregnancy. (**A**) For total choline, (**B**) for phosphatidylcholine, (**C**) for sphingomyelin, (**D**) for free choline, (**E**) for glycerophosphocholine, and (**F**) for phosphocholine.

Figure 1 shows the RCS curves for the associations of dietary choline intake in pregnancy with total CHD. The risk of total CHD in association with total choline, phosphatidylcholine, sphingomyelin, glycerophosphocholine, and phosphocholine plateaued after the intakes above 266.9 mg/d, 160.0 mg/d, 33.8 mg/d, 29.4 mg/d, and 25.5 mg/d, respectively. The percentages of controls exceeding the thresholds for total choline, phosphatidylcholine, sphingomyelin, glycerophosphocholine, and phosphocholine were 41.8%, 32.8%, 11.3%, 42.2%, and 11.0%, respectively. The RCS curves for the associations of total choline and choline derivatives with VSD and ASD showed similar shapes to those observed for total CHD (Figures S1 and S2).

The inverse associations between dietary choline intake and CHD appeared to be stronger for severe CHD compared to simple CHD; however, no statistically significant heterogeneity was observed (Table S7). The RCS curves depicting the associations of dietary choline intake with simple and severe CHD showed shapes comparable to those observed for total CHD (Figure S3).

### 3.3. Subgroup Analyses and Sensitivity Analyses

**Figure 2:**
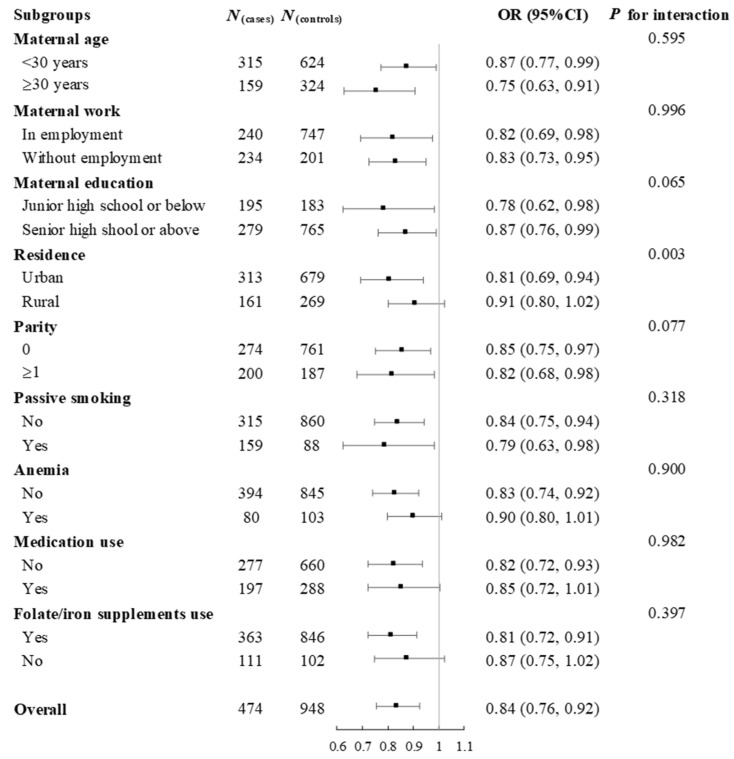
Subgroup analyses for the risk of total congenital heart defects associated with per 50 mg increase in total choline intake during pregnancy. Analyses were adjusted for total energy intake, maternal age, work, education, residence, parity, passive smoking, anemia, medication use, folate/iron supplement use, and dietary diversity score in pregnancy.

Subgroup analyses showed that the relationship between total choline intake during pregnancy and the risks of total CHD, VSD, and ASD remained largely unchanged across categories of maternal age, work, education, parity, passive smoking, anemia, medication use, or folate/iron supplements use in pregnancy (Figure 2, Figure S4 and Figure S5). However, the inverse associations appeared to be more pronounced among pregnant women in urban areas than those in rural areas, with *p* for interaction lower than 0.05. Sensitivity analyses showed that the risks of total CHD, VSD, and ASD associated with per 50 mg increase in total choline and choline derivatives did not change by using residual energy-adjusted intakes as exposures, additional adjustments for dietary intakes of other one-carbon metabolism nutrients including folate, vitamin B_12_, betaine, and methionine, or restricting analyses to mothers taking folate supplements in early pregnancy (Table S8).

## 4. Discussion

In this case–control study of Chinese pregnant women, we observed that higher maternal choline intake during pregnancy was associated with lower risks of CHD and its subtypes. The results for choline derivatives revealed a significantly inverse association with CHD for phosphatidylcholine, sphingomyelin, glycerophosphocholine, and phosphocholine during pregnancy, but not for free choline. Moreover, the inverse association between choline intake and CHD appeared to be stronger among pregnant women in urban areas. To our knowledge, this study represents the first investigation into the relationships between different choline derivatives in pregnancy and CHD.

Research focusing on the effect of choline on CHD remains limited to date. One study with 36 CHD cases and 41 normal controls reported a significant difference in choline concentration in maternal second and third trimester urine samples [14], while another study with 140 conotruncal cases and 280 normal controls observed no significant difference in maternal mid-pregnancy serum samples [15]. The inconsistent results may come from the discrepancy in sample size, CHD types, and the source and timing of maternal samples. Although choline concentration in serum and urine can be measured, its value as a standalone biomarker of habitual dietary intake is limited due to the metabolic factors [12]. To date, only one relevant study mentioned the association between dietary choline intake in pregnancy and CHD [16]. This study observed an inverse relationship for VSD among American pregnant women [16], in line with the results observed in the present study among Chinese pregnant women. Some human studies evaluated the relationship between choline and neural tube defects, a type of birth defect occurring in the first trimester, and a reduced risk was found to be associated with higher maternal choline intake and serum choline concentration [28,29]. No studies have evaluated pregnancy outcomes in association with different choline derivatives during pregnancy, making it difficult to compare the results about choline derivatives in our study with others. Further research is required to investigate the associations between choline derivatives and birth outcomes, including CHD.

One possible mechanism by which choline has an impact on CHD comes from the influence of homocysteine. Choline serves as a methyl donor in one-carbon metabolism to convert homocysteine to methionine [12]. Choline, obtained from the diet, is metabolized to betaine. Betaine then provides a methyl group to recycle homocysteine back into methionine, thereby lowering homocysteine levels and supporting the production of S-adenosylmethionine, a critical methyl donor for fetal development. Choline can also be used for synthesizing phosphatidylcholine, the main storage form of choline and an essential component of cell membranes, through the reaction that consumes S-adenosylmethionine and thus produces homocysteine [12]. Low choline intake in pregnancy may result in reduced choline concentration and further elevate homocysteine concentration [30]. The elevation of homocysteine due to low choline intake may also result in low levels of methyl donors for methylation reactions that are critical for heart development [31]. High homocysteine may further disturb methylation through its conversion to S-adenosylhomocysteine, a competitor of S-adenosylmethionine for methyltransferases [13]. In fact, high homocysteine concentration has been reported as an independent risk factor for CHD through the inhibition of DNA methyltransferase reactions, DNA hypomethylation, and altered gene expression [32]. Other possible mechanisms by which choline has an impact on CHD include reducing the production of pro-inflammatory cytokines and oxidative stress, supporting placental function, and interacting with other methyl donor nutrients involved in the one-carbon metabolism [33,34,35]. The absence of a significant association between free choline intake and CHD was a surprising finding. One potential explanation may lie in the gut microbiome-mediated metabolism of free choline. Evidence from other studies showed that free choline could be metabolized to trimethylamine-N-oxide, which may promote inflammatory and oxidative stress-related signaling pathways [36]. We hypothesize that the potential beneficial effects of free choline might have been offset by the production of trimethylamine-N-oxide. However, this remains speculative as we did not measure trimethylamine-N-oxide level or gut microbiota composition, and this hypothesis requires direct testing in future studies with relevant biomarkers. The stronger association observed between total choline intake and CHD risk in urban areas warrants further discussion. This heterogeneity may be attributed to several factors. First, dietary patterns between urban and rural areas differ markedly. Urban diets are typically richer in animal-source foods containing highly bioavailable forms of choline, such as phosphatidylcholine from eggs and sphingomyelin from meat, whereas rural diets may depend more on plant-based sources. As a result, urban pregnant women may have higher effective physiological choline exposure for the same reported intake. Second, socioeconomic disparities may play a role. Urban residents generally have higher education and income levels, which may contribute to more accurate dietary reporting and more consistent nutrient intake patterns, thereby reducing the likelihood of exposure misclassification.

Our study provides important evidence on CHD risk in relation to maternal choline intake during pregnancy. This study had several strengths, including the accurate diagnosis of CHD, the comprehensive assessment of maternal diet using a validated FFQ, the detailed adjustment for a wide range of potential confounders, and the exploration of associations across different choline subtypes. Nevertheless, some limitations merit discussion. First, selection bias may exist because this study did not recruit CHD fetuses that did not survive until birth. These excluded fetuses might be associated with more severe types of CHD and nutritional deficiencies, which could lead to an underestimation of the true strength of the association between choline intake and CHD. Selection bias may also come from the hospital-based design, in which participants may have a different diet from the general population, limiting the generalizability of our findings. Second, differential recall bias is a paramount concern in the case–control study. Mothers of infants with CHD may recall their diets differently from mothers of healthy infants, possibly due to emotional distress, despite evidence from prior studies suggesting that mothers could recall events in pregnancy well after years [37,38]. Underreporting of foods high in choline by case mothers could not be excluded, despite our efforts to minimize this bias, including standardized administration of a comprehensive FFQ and interviewer training to maintain neutrality. Third, exposure misclassification may exist because dietary data from the whole pregnancy, rather than the critical period of heart development in early pregnancy, were collected, although prior research indicated that maternal diets in pregnancy were overall stable throughout pregnancy [19]. This misalignment limits our ability to draw causal inferences about choline intake during the specific developmental window and CHD risk. Fourth, the lack of biological samples limits us from exploring the potential mechanisms for the associations. Fifth, the current sample size limits the ability to examine the relationships between maternal choline intake and other CHD subtypes. Finally, residual confounding bias may exist because of unobserved or unknown factors, and therefore, this study cannot establish a real causal relationship.

Our findings have several important implications. Clinically, prenatal care providers should emphasize the importance of adequate choline intake, which can be achieved through a diet rich in eggs, lean meat, and dairy products or through supplementation when dietary intake is insufficient. From a public health perspective, educational programs targeting pregnant women could highlight the critical role of choline in fetal heart development. Further studies are needed to determine the optimal dosage and timing of choline supplementation for the primary prevention of CHD.

## 5. Conclusions

In conclusion, this case–control study in the Chinese population suggests that higher maternal choline intake during pregnancy may reduce the risk of CHD. The significantly inverse association with CHD existed for maternal phosphatidylcholine, sphingomyelin, glycerophosphocholine, and phosphocholine intakes during pregnancy. These findings provide preliminary, hypothesis-generating evidence of the association between maternal choline intake and CHD. However, given the limitations of this case–control study, future verification is needed through more robustly designed studies, such as prospective cohort studies, to confirm the observed associations and evaluate their potential for informing primary prevention strategies for fetal CHD. Furthermore, studies integrating different choline derivatives and biological markers are needed to validate these findings and clarify underlying mechanisms.

## Figures and Tables

**Table 1:** Characteristics of the study participants among cases and controls.

	Cases (*N* = 474)	Controls (*N* = 948)	*p* ^1^
Sociodemographic characteristics, n (%)			
Maternal age ≥ 30 years	159 (33.5)	324 (34.2)	0.812
Maternal work, in employment	240 (50.6)	747 (78.8)	<0.001
Maternal education, senior high school or above	279 (58.9)	765 (80.7)	<0.001
Rural residence	161 (34.0)	269 (28.4)	0.030
Nulliparity	274 (57.8)	761 (80.3)	<0.001
Maternal health-related factors in pregnancy, n (%)			
Passive smoking	159 (33.5)	88 (9.3)	<0.001
Anemia	80 (16.9)	103 (10.9)	0.001
Medication use	197 (41.6)	288 (30.4)	<0.001
Folate/iron supplements use	363 (76.6)	846 (89.2)	<0.001
Dietary choline intake in pregnancy, median (25th percentile, 75th percentile), mg/d			
Total choline	190.4 (120.1, 256.7)	246.9 (188.1, 322.2)	<0.001
Phosphatidylcholine	89.9 (49.1, 145.5)	139.3 (91.3, 170.6)	<0.001
Sphingomyelin	10.2 (6.1, 17.5)	15.1 (10.9, 22.1)	<0.001
Free choline	48.1 (33.2, 65.0)	54.6 (36.9, 79.9)	<0.001
Glycerophosphocholine	17.5 (9.0, 27.6)	26.4 (17.9, 36.8)	<0.001
Phosphocholine	9.4 (5.5, 14.3)	13.6 (9.1, 18.9)	<0.001

^1^ *p*-values are from χ^2^ test for categorical variables and from Mann–Whitney U test for continuous variables.

**Table 2:** Associations of dietary choline intake during pregnancy with total congenital heart defects.

Dietary Choline	Tertiles of Dietary Choline Intake, OR (95%CI)			*p* for Trend	Per 50 mg Increase
	Tertile 1	Tertile 2	Tertile 3		
Total choline					
Intake, mg/d	<205.14	205.14–290.08	>290.08		
*N*_(cases)_/*N*_(controls)_	268/316	121/315	85/317		
Model 1	1	0.42 (0.32, 0.56)	0.26 (0.17, 0.40)	<0.001	0.73 (0.66, 0.80)
Model 2	1	0.50 (0.37, 0.68)	0.37 (0.23, 0.57)	<0.001	0.82 (0.74, 0.90)
Model 3	1	0.53 (0.38, 0.73)	0.38 (0.24, 0.61)	<0.001	0.84 (0.76, 0.92)
Phosphatidylcholine					
Intake, mg/d	<110.81	110.81–159.39	>159.39		
*N*_(cases)_/*N*_(controls)_	273/316	121/316	80/316		
Model 1	1	0.30 (0.21, 0.43)	0.45 (0.34, 0.58)	<0.001	0.63 (0.55, 0.72)
Model 2	1	0.41 (0.28, 0.61)	0.51 (0.38, 0.69)	<0.001	0.72 (0.63, 0.83)
Model 3	1	0.41 (0.27, 0.61)	0.51 (0.38, 0.70)	<0.001	0.72 (0.63, 0.83)
Sphingomyelin					
Intake, mg/d	<12.22	12.22–18.92	>18.92		
*N*_(cases)_/*N*_(controls)_	279/315	83/316	112/317		
Model 1	1	0.45 (0.33, 0.63)	0.31 (0.23, 0.42)	<0.001	0.43 (0.24, 0.76)
Model 2	1	0.54 (0.38, 0.77)	0.34 (0.25, 0.47)	<0.001	0.59 (0.36, 0.95)
Model 3	1	0.59 (0.40, 0.86)	0.37 (0.26, 0.51)	<0.001	0.59 (0.36, 0.96)
Free choline					
Intake, mg/d	<42.60	42.60–70.10	>70.10		
*N*_(cases)_/*N*_(controls)_	192/316	180/315	102/317		
Model 1	1	1.13 (0.85, 1.52)	0.83 (0.54, 1.28)	0.571	1.03 (0.69, 1.54)
Model 2	1	1.25 (0.92, 1.71)	0.86 (0.55, 1.35)	0.786	1.01 (0.66, 1.54)
Model 3	1	1.31 (0.94, 1.81)	0.94 (0.59, 1.51)	0.927	1.06 (0.68, 1.66)
Glycerophosphocholine					
Intake, mg/d	>20.86	20.86–32.85	>32.85		
*N*_(cases)_/*N*_(controls)_	280/315	120/316	74/317		
Model 1	1	0.39 (0.30, 0.52)	0.21 (0.14, 0.31)	<0.001	0.05 (0.03, 0.10)
Model 2	1	0.52 (0.38, 0.70)	0.32 (0.21, 0.51)	<0.001	0.14 (0.07, 0.29)
Model 3	1	0.54 (0.40, 0.75)	0.34 (0.21, 0.53)	<0.001	0.15 (0.07, 0.31)
Phosphocholine					
Intake, mg/d	<10.22	10.22–16.38	>16.38		
*N*_(cases)_/*N*_(controls)_	261/315	119/316	94/317		
Model 1	1	0.45 (0.34, 0.60)	0.35 (0.24, 0.52)	<0.001	0.05 (0.02, 0.16)
Model 2	1	0.54 (0.40, 0.74)	0.46 (0.30, 0.69)	<0.001	0.13 (0.04, 0.41)
Model 3	1	0.59 (0.43, 0.82)	0.53 (0.34, 0.82)	0.001	0.16 (0.05, 0.49)

Model 1 was adjusted for total energy intake in pregnancy. Model 2 was adjusted for total energy intake in pregnancy, maternal age, work, education, residence, and parity. Model 3 was adjusted for all factors in Model 2, plus maternal passive smoking, anemia, medication use, folate/iron supplement use, and dietary diversity score in pregnancy.

**Table 3:** Associations of choline intakes from lipid- and water-soluble sources during pregnancy with total congenital heart defects.

Dietary Choline	Tertiles of Dietary Choline Intake, OR (95%CI)			*p* for Trend	Per 50 mg Increase
	Tertile 1	Tertile 2	Tertile 3		
Lipid-soluble dietary choline					
Intake, mg/d	<126.62	126.62–178.49	>178.49		
*N*_(cases)_/*N*_(controls)_	272/316	122/316	80/316		
Model 1	1	0.45 (0.34, 0.59)	0.29 (0.20, 0.42)	<0.001	0.65 (0.58, 0.73)
Model 2	1	0.52 (0.39, 0.70)	0.39 (0.26, 0.58)	<0.001	0.74 (0.65, 0.83)
Model 3	1	0.53 (0.39, 0.72)	0.40 (0.27, 0.61)	<0.001	0.74 (0.65, 0.84)
Water-soluble dietary choline					
Intake, mg/d	<75.89	75.89–118.70	>118.70		
*N*_(cases)_/*N*_(controls)_	239/315	148/316	87/317		
Model 1	1	0.60 (0.45, 0.79)	0.33 (0.21, 0.52)	<0.001	0.55 (0.42, 0.71)
Model 2	1	0.70 (0.51, 0.95)	0.44 (0.28, 0.70)	0.001	0.65 (0.49, 0.85)
Model 3	1	0.78 (0.56, 1.07)	0.51 (0.31, 0.84)	0.009	0.68 (0.51, 0.89)

Model 1 was adjusted for total energy intake in pregnancy. Model 2 was adjusted for total energy intake in pregnancy, maternal age, work, education, residence, and parity. Model 3 was adjusted for all factors in Model 2, plus maternal passive smoking, anemia, medication use, folate/iron supplement use, and dietary diversity score in pregnancy.

## Data Availability

The original contributions presented in this study are included in the article/Supplementary Materials. Further inquiries can be directed to the corresponding author.

## Supplementary Materials

The following supporting information can be downloaded at: https://www.mdpi.com/article/10.3390/nu18010126/s1, Table S1: Spearman correlation matrix of dietary choline derivatives intake among the study participants; Table S2: The constitution of congenital heart defects subtypes in the cases; Table S3: Characteristics of the study participants according to tertiles of total choline intake during pregnancy; Table S4: Associations of dietary choline intake during pregnancy with ventricular septal defects; Table S5: Associations of dietary choline intake during pregnancy with atrial septal defects; Table S6: Associations of choline intakes from lipid- and water-soluble sources during pregnancy with ventricular septal defects and atrial septal defects; Table S7: Associations of total dietary choline intake during pregnancy with simple and severe congenital heart defects; Table S8: Sensitivity analyses for the risk of congenital heart defects associated with per 50 mg increase in choline intake during pregnancy; Figure S1: Restricted cubic spline models for the associations of dietary choline intake during pregnancy with ventricular septal defects; Figure S2: Restricted cubic spline models for the associations of dietary choline intake during pregnancy with atrial septal defects; Figure S3: Restricted cubic spline models for the associations of total dietary choline intake during pregnancy with simple and severe congenital heart defects; Figure S4: Subgroup analyses for the risk of ventricular septal defects associated with per 50 mg increase in total choline intake during pregnancy; Figure S5: Subgroup analyses for the risk of atrial septal defects associated with per 50 mg increase in total choline intake during pregnancy.

## Abbreviations

The following abbreviations are used in this manuscript:

ASD	Atrial septal defects
CHD	Congenital heart defects
RCS	Restricted cubic spline
VSD	Ventricular septal defects
