# Supplementary material for: Dietary Choline Intake During Pregnancy and Congenital Heart Defects in a Chinese Population
Source: Nutrients. 2025 Dec 31;18(1):126. doi: 10.3390/nu18010126 (PMC12788036; doi:10.3390/nu18010126)
Supplement: Supplementary file 1 [file nutrients-18-00126-s001.zip › nutrients-3984681-supplementary.pdf]

Table S1. Spearman correlation matrix of dietary choline derivatives intake among the study participants.

	Phosphatidylcholine	Sphingomyelin	Free choline	Glycerophosphorylcholine	Phosphorylcholine
Phosphatidylcholine	1.00				
Sphingomyelin	0.69	1.00			
Free choline	0.62	0.63	1.00		
Glycerophosphorylcholine	0.63	0.70	0.74	1.00	
Phosphorylcholine	0.62	0.83	0.86	0.85	1.00

Dietary choline derivatives were strongly positively correlated with each other, with all $P < 0.001$.

Table S2. The constitution of congenital heart defects subtypes in the cases.

Subtypes	Number	Proportion, %
Ventricular heart defects	222	46.84
Atrial septal defects	218	45.99
Atrioventricular septal defects	87	18.35
Patent ductus arteriosus	74	15.61
Tetralogy of fallot	29	6.12
Pulmonary stenosis	17	3.59
Aortic valve stenosis	14	2.95
Transposition of the great arteries	7	1.48
Single ventricle	5	1.05
Others	2	0.42

A fetus with congenital heart defects may be diagnosed with two or more subtypes of congenital heart defects.

Table S3. Characteristics of the study participants according to tertiles of total choline intake during pregnancy.

	Cases (N = 474)				Controls (N = 948)			
	Tertile 1 (N = 268)	Tertile 2 (N = 121)	Tertile 3 (N = 85)	P[1]	Tertile 1 (N = 316)	Tertile 2 (N = 315)	Tertile 3 (N = 317)	P[1]
Sociodemographic characteristics, n (%)								
Maternal age ≥30 years	90 (33.6)	44 (36.4)	25 (29.4)	0.582	113 (35.8)	105 (33.3)	106 (33.4)	0.768
Maternal work, in employment	120 (44.8)	71 (58.7)	49 (57.7)	0.014	238 (75.3)	255 (80.9)	254 (80.1)	0.174
Maternal education, senior high school or above	136 (50.8)	87 (71.9)	56 (65.9)	<0.001	247(78.2)	248 (78.7)	270 (85.2)	0.043
Rural residence	68 (25.4)	54 (44.6)	39 (45.9)	<0.001	105 (33.2)	97 (30.8)	67 (21.1)	0.002
Nullparity	143 (53.4)	72 (59.5)	59 (69.4)	0.030	251 (79.4)	243 (77.1)	267 (84.2)	0.074
Maternal health-related factors in pregnancy, n (%)								
Passive smoking	94 (35.1)	39 (32.2)	26 (30.6)	0.702	38 (12.0)	27 (8.6)	23 (7.3)	0.102
Anemia	48 (17.9)	24 (19.8)	8 (9.4)	0.114	41 (13.0)	29 (9.2)	33 (10.4)	0.299
Medication use	111 (41.4)	54 (44.6)	32 (37.7)	0.604	95 (30.1)	84 (26.7)	109 (34.4)	0.107
Folate/iron supplements use	210 (78.4)	93 (76.9)	60 (70.6)	0.336	292 (92.4)	283 (89.8)	271 (85.5)	0.018
Dietary choline intake in pregnancy, median (25th percentile, 75th percentile), mg/d								
Total choline	131.0 (90.9, 164.9)	240.9 (224.0, 258.3)	351.3 (322.6, 421.0)	<0.001	154.9 (122.3, 188.1)	246.8 (225.8, 267.0)	364.6 (321.7, 430.5)	<0.001
Phosphatidylcholine	56.1 (35.1, 82.0)	140.2 (119.4, 153.0)	184.3 (169.2, 219.9)	<0.001	76.3 (52.4, 97.3)	141.5 (122.4, 153.0)	184.3 (168.5, 215.3)	<0.001
Sphingomyelin	6.8 (4.2, 10.0)	13.8 (10.3, 17.5)	27.2 (19.0, 41.5)	<0.001	10.2 (7.6, 13.1)	15.2 (11.9, 18.5)	25.6 (18.6, 38.5)	<0.001
Free choline	36.9 (28.0, 49.0)	53.9 (45.8, 65.4)	87.2 (71.6, 110.1)	<0.001	33.5 (26.6, 44.1)	53.4 (41.4, 66.7)	90.0 (73.0, 110.4)	<0.001
Glycerophosphocholine	10.6 (6.9, 16.9)	23.3 (18.2, 29.2)	35.6 (29.1, 51.3)	<0.001	16.3 (11.8, 21.7)	25.3 (20.3, 31.7)	39.5 (33.1, 48.1)	<0.001
Phosphocholine	6.2 (4.3, 9.4)	11.4 (9.1, 14.3)	20.4 (15.7, 29.0)	<0.001	8.4 (6.6, 10.6)	13.1 (10.1, 15.6)	22.3 (17.5, 28.9)	<0.001

[1] P values are from χ^2 test for categorical variables and from Kruskal-Wallis test for continuous variables.

Table S4. Associations of dietary choline intake during pregnancy with ventricular septal defects.

Dietary choline	Tertiles of dietary choline intake, OR (95%CI)			*P* for trend	Per 50 mg increase
	Tertile 1	Tertile 2	Tertile 3		
Total choline					
Intake, mg/d	<205.14	205.14-290.08	>290.08		
$N_{(cases)}/N_{(controls)}$	127/316	55/315	40/317		
Model 1	1	0.39 (0.26, 0.58)	0.22 (0.13, 0.40)	<0.001	0.68 (0.60, 0.77)
Model 2	1	0.48 (0.32, 0.73)	0.32 (0.18, 0.59)	<0.001	0.79 (0.70, 0.90)
Model 3	1	0.53 (0.34, 0.80)	0.35 (0.19, 0.66)	<0.001	0.81 (0.71, 0.93)
Phosphatidylcholine					
Intake, mg/d	<110.81	110.81-159.39	>159.39		
$N_{(cases)}/N_{(controls)}$	131/316	54/316	37/316		
Model 1	1	0.41 (0.29, 0.59)	0.26 (0.16, 0.43)	<0.001	0.60 (0.50, 0.71)
Model 2	1	0.51 (0.34, 0.76)	0.38 (0.22, 0.65)	<0.001	0.71 (0.59, 0.85)
Model 3	1	0.51 (0.34, 0.76)	0.37 (0.22, 0.64)	<0.001	0.71 (0.60, 0.85)
Sphingomyelin					
Intake, mg/d	<12.22	12.22-18.92	>18.92		
$N_{(cases)}/N_{(controls)}$	137/315	33/316	52/317		
Model 1	1	0.39 (0.25, 0.61)	0.24 (0.16, 0.37)	<0.001	0.18 (0.07, 0.49)
Model 2	1	0.49 (0.31, 0.78)	0.27 (0.18, 0.42)	<0.001	0.32 (0.13, 0.79)
Model 3	1	0.51 (0.31, 0.83)	0.29 (0.18, 0.45)	<0.001	0.31 (0.13, 0.78)
Free choline					
Intake, mg/d	<42.60	42.60-70.10	>70.10		
$N_{(cases)}/N_{(controls)}$	91/316	83/315	48/317		
Model 1	1	1.06 (0.72, 1.56)	0.73 (0.41, 1.32)	0.402	0.97 (0.56, 1.66)
Model 2	1	1.21 (0.80, 1.82)	0.76 (0.42, 1.38)	0.538	0.94 (0.53, 1.66)
Model 3	1	1.22 (0.79, 1.86)	0.88 (0.47, 1.64)	0.868	1.01 (0.56, 1.83)
Glycerophosphocholine					
Intake, mg/d	>20.86	20.86-32.85	>32.85		
$N_{(cases)}/N_{(controls)}$	128/315	57/316	37/317		
Model 1	1	0.42 (0.29, 0.60)	0.23 (0.14, 0.40)	<0.001	0.03 (0.01, 0.09)
Model 2	1	0.54 (0.36, 0.82)	0.35 (0.19, 0.62)	<0.001	0.11 (0.04, 0.29)
Model 3	1	0.57 (0.38, 0.86)	0.36 (0.20, 0.64)	<0.001	0.12 (0.04, 0.32)
Phosphocholine					
Intake, mg/d	<10.22	10.22-16.38	>16.38		
$N_{(cases)}/N_{(controls)}$	125/315	53/316	44/317		
Model 1	1	0.40 (0.27, 0.59)	0.30 (0.17, 0.51)	<0.001	0.02 (0.01, 0.03)
Model 2	1	0.50 (0.33, 0.75)	0.41 (0.23, 0.71)	<0.001	0.04 (0.01, 0.20)
Model 3	1	0.57 (0.38, 0.87)	0.48 (0.27, 0.85)	0.005	0.05 (0.01, 0.28)

Model 1 was adjusted for total energy intake in pregnancy. Model 2 was adjusted for total energy intake in pregnancy, maternal age, work, education, residence, and parity. Model 3 was adjusted for all factors in Model 2 plus maternal passive smoking, anemia, medication use, folate/iron supplements use, and dietary diversity score in pregnancy.

Table S5. Associations of dietary choline intake during pregnancy with atrial septal defects.

Dietary choline	Tertiles of dietary choline intake, OR (95%CI)			*P* for trend	Per 50 mg increase
	Tertile 1	Tertile 2	Tertile 3		
Total choline					
Intake, mg/d	<205.14	205.14-290.08	>290.08		
$N_{(cases)}/N_{(controls)}$	114/316	58/315	46/317		
Model 1	1	0.44 (0.30, 0.66)	0.28 (0.16, 0.50)	<0.001	0.73 (0.65, 0.83)
Model 2	1	0.49 (0.33, 0.73)	0.38 (0.21, 0.68)	<0.001	0.81 (0.71, 0.92)
Model 3	1	0.53 (0.35, 0.81)	0.39 (0.22, 0.72)	0.001	0.81 (0.71, 0.92)
Phosphatidylcholine					
Intake, mg/d	<110.81	110.81-159.39	>159.39		
$N_{(cases)}/N_{(controls)}$	118/316	57/316	43/316		
Model 1	1	0.47 (0.33, 0.67)	0.33 (0.20, 0.53)	<0.001	0.66 (0.56, 0.78)
Model 2	1	0.52 (0.35, 0.77)	0.42 (0.25, 0.70)	<0.001	0.73 (0.62, 0.87)
Model 3	1	0.52 (0.35, 0.77)	0.42 (0.25, 0.71)	<0.001	0.73 (0.62, 0.87)
Sphingomyelin					
Intake, mg/d	<12.22	12.22-18.92	>18.92		
$N_{(cases)}/N_{(controls)}$	112/315	46/316	60/317		
Model 1	1	0.58 (0.37, 0.90)	0.42 (0.29, 0.62)	0.003	0.47 (0.27, 0.81)
Model 2	1	0.65 (0.41, 1.03)	0.43 (0.28, 0.64)	0.016	0.64 (0.48, 0.85)
Model 3	1	0.67 (0.42, 1.09)	0.44 (0.29, 0.67)	0.03	0.53 (0.35, 0.82)
Free choline					
Intake, mg/d	<42.60	42.60-70.10	>70.10		
$N_{(cases)}/N_{(controls)}$	82/316	84/315	52/317		
Model 1	1	1.14 (0.77, 1.68)	0.80 (0.45, 1.44)	0.592	0.94 (0.55, 1.59)
Model 2	1	1.17 (0.77, 1.75)	0.77 (0.42, 1.41)	0.536	0.92 (0.53, 1.59)
Model 3	1	1.20 (0.79, 1.83)	0.86 (0.46, 1.60)	0.794	0.97 (0.55, 1.70)
Glycerophosphocholine					
Intake, mg/d	>20.86	20.86-32.85	>32.85		
$N_{(cases)}/N_{(controls)}$	123/315	58/316	37/317		
Model 1	1	0.39 (0.27, 0.58)	0.18 (0.11, 0.32)	<0.001	0.04 (0.02, 0.11)
Model 2	1	0.49 (0.33, 0.73)	0.27 (0.15, 0.49)	<0.001	0.10 (0.04, 0.25)
Model 3	1	0.52 (0.34, 0.77)	0.28 (0.15, 0.51)	<0.001	0.10 (0.04, 0.26)
Phosphocholine					
Intake, mg/d	<10.22	10.22-16.38	>16.38		
$N_{(cases)}/N_{(controls)}$	112/315	53/316	53/317		
Model 1	1	0.45 (0.30, 0.66)	0.41 (0.24, 0.70)	<0.001	0.04 (0.01, 0.19)
Model 2	1	0.50 (0.29, 0.86)	0.49 (0.33, 0.74)	0.004	0.10 (0.02, 0.47)
Model 3	1	0.55 (0.31, 0.96)	0.54 (0.35, 0.82)	0.015	0.10 (0.02, 0.47)

Model 1 was adjusted for total energy intake in pregnancy. Model 2 was adjusted for total energy intake in pregnancy, maternal age, work, education, residence, and parity. Model 3 was adjusted for all factors in Model 2 plus maternal passive smoking, anemia, medication use, folate/iron supplements use, and dietary diversity score in pregnancy.

Table S6. Associations of choline intakes from lipid- and water-soluble sources during pregnancy with ventricular septal defects and atrial septal defects.

	Tertiles of dietary choline intake, OR (95%CI)			*P* for trend	Per 50 mg increase
	Tertile 1	Tertile 2	Tertile 3		
Ventricular septal defects					
Lipid-soluble dietary choline					
Intake, mg/d	<126.62	126.62-178.49	>178.49		
$N_{(cases)}/N_{(controls)}$	129/316	56/316	37/316		
Model 1	1	0.43 (0.30, 0.62)	0.26 (0.16, 0.43)	<0.001	0.61 (0.51, 0.71)
Model 2	1	0.54 (0.37, 0.79)	0.37 (0.22, 0.64)	<0.001	0.71 (0.60, 0.84)
Model 3	1	0.54 (0.36, 0.81)	0.37 (0.21, 0.65)	<0.001	0.71 (0.60, 0.84)
Water-soluble dietary choline					
Intake, mg/d	<75.89	75.89-118.70	>118.70		
$N_{(cases)}/N_{(controls)}$	113/315	72/316	37/317		
Model 1	1	0.56 (0.38, 0.81)	0.22 (0.12, 0.42)	<0.001	0.48 (0.34, 0.68)
Model 2	1	0.66 (0.44, 0.99)	0.30 (0.15, 0.57)	<0.001	0.58 (0.41, 0.84)
Model 3	1	0.76 (0.50, 1.15)	0.34 (0.17, 0.67)	0.004	0.62 (0.43, 0.90)
Atrial septal defects					
Lipid-soluble dietary choline					
Intake, mg/d	<126.62	126.62-178.49	>178.49		
$N_{(cases)}/N_{(controls)}$	117/316	56/316	45/316		
Model 1	1	0.46 (0.32, 0.67)	0.34 (0.21, 0.56)	<0.001	0.68 (0.58, 0.80)
Model 2	1	0.51 (0.35, 0.75)	0.43 (0.26, 0.72)	<0.001	0.75 (0.64, 0.88)
Model 3	1	0.51 (0.35, 0.76)	0.43 (0.26, 0.73)	<0.001	0.75 (0.64, 0.88)
Water-soluble dietary choline					
Intake, mg/d	<75.89	75.89-118.70	>118.70		
$N_{(cases)}/N_{(controls)}$	103/315	66/316	49/317		
Model 1	1	0.58 (0.39, 0.86)	0.37 (0.20, 0.68)	0.001	0.52 (0.37, 0.73)
Model 2	1	0.63 (0.42, 0.95)	0.45 (0.24, 0.84)	0.008	0.61 (0.43, 0.86)
Model 3	1	0.67 (0.44, 1.02)	0.50 (0.26, 0.95)	0.026	0.61 (0.43, 0.87)

Model 1 was adjusted for total energy intake in pregnancy. Model 2 was adjusted for total energy intake in pregnancy, maternal age, work, education, residence, and parity. Model 3 was adjusted for all factors in Model 2 plus maternal passive smoking, anemia, medication use, folate/iron supplements use, and dietary diversity score in pregnancy.

Table S7. Associations of total dietary choline intake during pregnancy with simple and severe congenital heart defects.

	Tertiles of total dietary choline intake, OR (95%CI)			*P* for trend	Per 50 mg increase
	Tertile 1	Tertile 2	Tertile 3		
Simple congenital heart defects					
$N_{(cases)}/N_{(controls)}$	166/316	79/315	66/317		
Model 1	1	0.46 (0.33, 0.65)	0.36 (0.23, 0.59)	<0.001	0.75 (0.68, 0.84)
Model 2	1	0.52 (0.38, 0.72)	0.38 (0.24, 0.61)	<0.001	0.85 (0.76, 0.95)
Model 3	1	0.54 (0.38, 0.76)	0.46 (0.32, 0.66)	0.004	0.86 (0.81, 0.92)
Severe congenital heart defects					
$N_{(cases)}/N_{(controls)}$	102/316	42/315	19/317		
Model 1	1	0.34 (0.22, 0.53)	0.11 (0.04, 0.27)	<0.001	0.65 (0.56, 0.75)
Model 2	1	0.40 (0.26, 0.64)	0.14 (0.06, 0.29)	<0.001	0.73 (0.62, 0.85)
Model 3	1	0.43 (0.27, 0.68)	0.14 (0.06, 0.31)	<0.001	0.74 (0.63, 0.86)

Model 1 was adjusted for total energy intake in pregnancy. Model 2 was adjusted for total energy intake in pregnancy, maternal age, work, education, residence, and parity. Model 3 was adjusted for all factors in Model 2 plus maternal passive smoking, anemia, medication use, folate/iron supplements use, and dietary diversity score in pregnancy.

Table S8. Sensitivity analyses for the risk of congenital heart defects associated with per 50 mg increase in choline intake during pregnancy.

	OR (95%CI)					
	Using the residual energy-adjusted intakes as exposures	Additional adjustment for dietary folate	Additional adjustment for dietary vitamin B_{12}	Additional adjustment for dietary betaine	Additional adjustment for dietary methionine	Restricting to mothers taking folate supplements in early pregnancy
Total congenital heart defects						
Per 50 mg increase in total choline	0.82 (0.74, 0.90)	0.86 (0.77, 0.97)	0.85 (0.78, 0.92)	0.84 (0.75, 0.90)	0.83 (0.75, 0.89)	0.87 (0.77, 0.98)
Per 50 mg increase in phosphatidylcholine	0.72 (0.63, 0.83)	0.74 (0.64, 0.85)	0.72 (0.68, 0.77)	0.74 (0.64, 0.83)	0.71 (0.63, 0.82)	0.77 (0.66, 0.91)
Per 50 mg increase in sphingomyelin	0.56 (0.32, 0.97)	0.57 (0.33, 0.97)	0.56 (0.37, 0.85)	0.58 (0.33, 0.97)	0.56 (0.33, 0.96)	0.57 (0.35, 0.92)
Per 50 mg increase in free choline	1.06 (0.68, 1.66)	1.08 (0.69, 1.69)	1.06 (0.73, 1.55)	1.08 (0.69, 1.66)	1.07 (0.70, 1.64)	1.12 (0.71, 1.79)
Per 50 mg increase in glycerophosphocholine	0.15 (0.08, 0.27)	0.16 (0.11, 0.25)	0.15 (0.20, 0.11)	0.17 (0.16, 0.31)	0.15 (0.16, 0.14)	0.15 (0.08, 0.30)
Per 50 mg increase in phosphocholine	0.16 (0.05, 0.49)	0.17 (0.06, 0.49)	0.16 (0.10, 0.24)	0.18 (0.06, 0.49)	0.16 (0.06, 0.41)	0.16 (0.05, 0.56)
Ventricular septal defects						
Per 50 mg increase in total choline	0.79 (0.70, 0.90)	0.84 (0.72, 0.97)	0.82 (0.74, 0.90)	0.81 (0.71, 0.90)	0.79 (0.70, 0.89)	0.84 (0.71, 0.99)
Per 50 mg increase in phosphatidylcholine	0.71 (0.71, 0.85)	0.73 (0.72, 0.73)	0.71 (0.76, 0.67)	0.73 (0.72, 0.85)	0.71 (0.72, 0.70)	0.77 (0.74, 0.81)
Per 50 mg increase in sphingomyelin	0.31 (0.13, 0.78)	0.33 (0.14, 0.79)	0.31 (0.18, 0.56)	0.33 (0.14, 0.78)	0.32 (0.15, 0.72)	0.37 (0.15, 0.91)
Per 50 mg increase in free choline	1.01 (0.56, 1.83)	1.03 (0.57, 1.85)	1.01 (0.61, 1.68)	1.03 (0.57, 1.83)	1.01 (0.57, 1.80)	1.07 (0.59, 1.96)
Per 50 mg increase in glycerophosphocholine	0.12 (0.04, 0.32)	0.13 (0.05, 0.33)	0.12 (0.09, 0.14)	0.14 (0.05, 0.32)	0.12 (0.05, 0.26)	0.18 (0.07, 0.45)
Per 50 mg increase in phosphocholine	0.05 (0.01, 0.28)	0.06 (0.02, 0.22)	0.05 (0.06, 0.04)	0.07 (0.02, 0.28)	0.05 (0.02, 0.13)	0.07 (0.04, 0.12)
Atrial septal defects						
Per 50 mg increase in total choline	0.76 (0.65, 0.90)	0.82 (0.69, 0.96)	0.81 (0.69, 0.95)	0.78 (0.66, 0.90)	0.76 (0.66, 0.88)	0.82 (0.67, 0.98)
Per 50 mg increase in phosphatidylcholine	0.73 (0.61, 0.88)	0.75 (0.62, 0.91)	0.73 (0.66, 0.82)	0.75 (0.62, 0.88)	0.74 (0.63, 0.87)	0.79 (0.63, 0.99)
Per 50 mg increase in sphingomyelin	0.57 (0.36, 0.92)	0.59 (0.37, 0.94)	0.57 (0.41, 0.81)	0.59 (0.37, 0.92)	0.58 (0.37, 0.90)	0.60 (0.36, 0.96)
Per 50 mg increase in free choline	0.97 (0.53, 1.76)	0.98 (0.54, 1.79)	0.97 (0.58, 1.61)	0.99 (0.54, 1.76)	0.97 (0.54, 1.73)	1.03 (0.56, 1.89)
Per 50 mg increase in glycerophosphocholine	0.10 (0.03, 0.28)	0.12 (0.04, 0.31)	0.10 (0.03, 0.28)	0.11 (0.04, 0.32)	0.11 (0.04, 0.27)	0.12 (0.04, 0.36)
Per 50 mg increase in phosphocholine	0.10 (0.02, 0.47)	0.11 (0.03, 0.37)	0.10 (0.02, 0.47)	0.11 (0.03, 0.38)	0.12 (0.05, 0.29)	0.11 (0.03, 0.44)
Simple congenital heart defects						
Per 50 mg increase in total choline	0.86 (0.80, 0.92)	0.88 (0.82, 0.95)	0.87 (0.81, 0.94)	0.89 (0.81, 0.97)	0.87 (0.81, 0.94)	0.89 (0.82, 0.97)
Severe congenital heart defects						
Per 50 mg increase in total choline	0.74 (0.63, 0.87)	0.74 (0.64, 0.87)	0.75 (0.64, 0.87)	0.76 (0.65, 0.88)	0.73 (0.62, 0.86)	0.74 (0.63, 0.87)

Analyses were minimally adjusted for total energy intake, maternal age, work, education, residence, parity, passive smoking, anemia, medication use, folate/iron supplements use, and dietary diversity score in pregnancy.

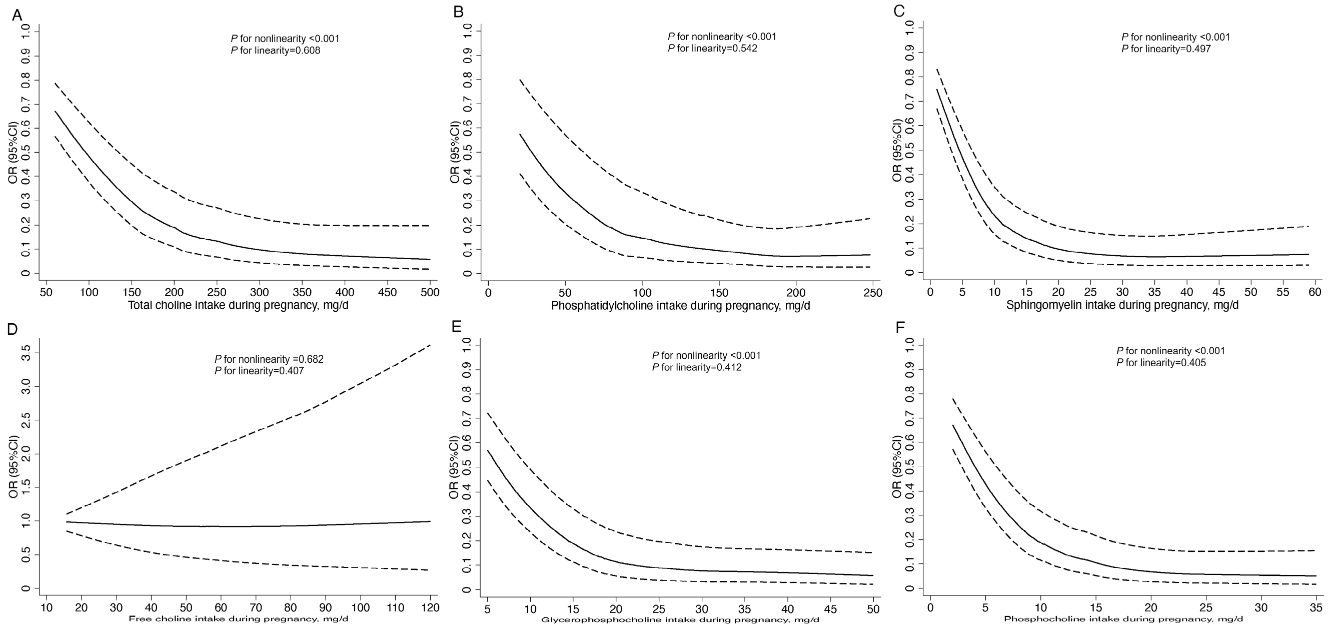

Figure S1. Restricted cubic spline models for the associations of dietary choline intake during pregnancy with ventricular septal defects. Analyses were adjusted for total energy intake, maternal age, work, education, residence, parity, passive smoking, anemia, medication use, folate/iron supplements use, and dietary diversity score in pregnancy. (A) for total choline, (B) for phosphatidylcholine, (C) for sphingomyelin, (D) for free choline, (E) for glycerophosphocholine, and (F) for phosphocholine.

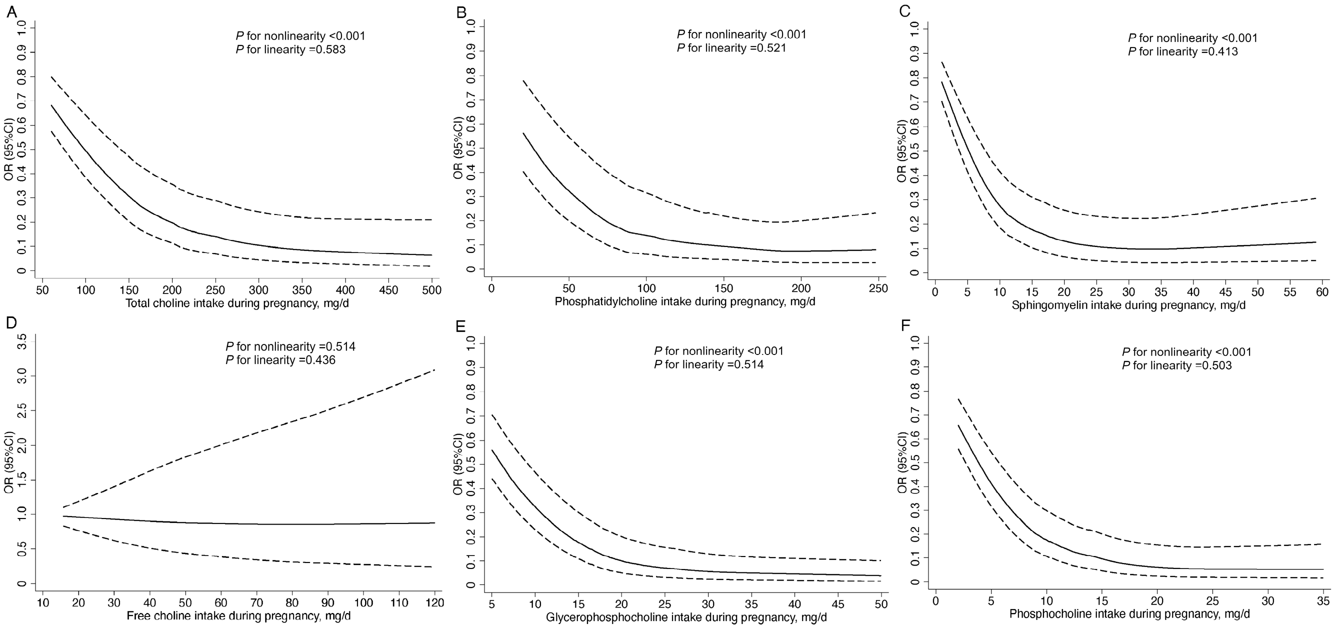

Figure S2. Restricted cubic spline models for the associations of dietary choline intake during pregnancy with atrial septal defects. Analyses were adjusted for total energy intake, maternal age, work, education, residence, parity, passive smoking, anemia, medication use, folate/iron supplements use, and dietary diversity score in pregnancy. (A) for total choline, (B) for phosphatidylcholine, (C) for sphingomyelin, (D) for free choline, (E) for glycerophosphocholine, and (F) for phosphocholine.

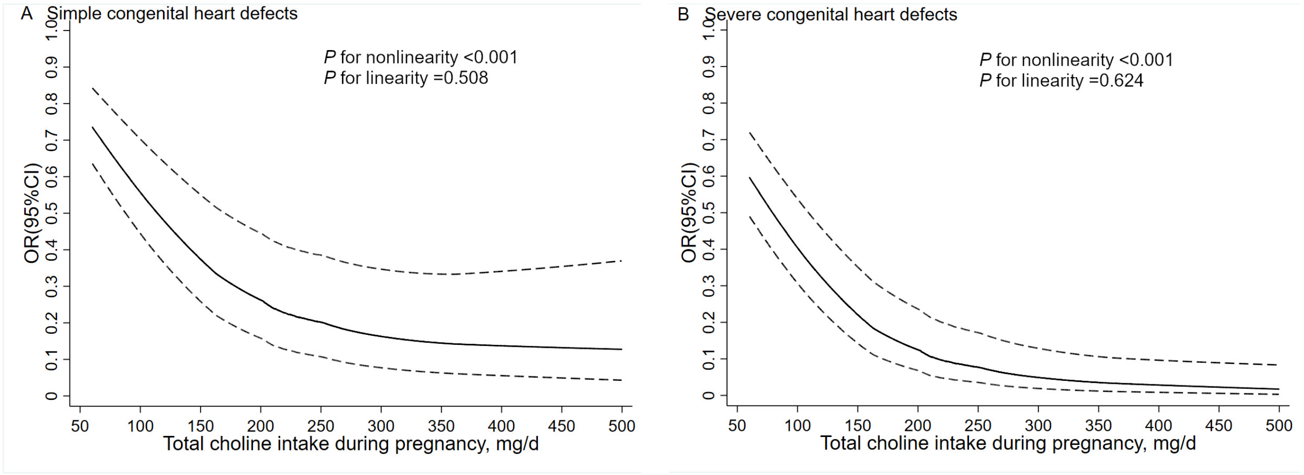

Figure S3. Restricted cubic spline models for the associations of total dietary choline intake during pregnancy with simple and severe congenital heart defects. Analyses were adjusted for total energy intake, maternal age, work, education, residence, parity, passive smoking, anemia, medication use, folate/iron supplements use, and dietary diversity score in pregnancy. (A) for simple congenital heart defects, and (B) for severe congenital heart defects.

Subgroups	$N_{(cases)}$	$N_{(controls)}$	OR (95%CI)	*P* for interaction
**Maternal age**				0.626
<30 years	150	624	0.85 (0.72, 1.01)	
≥ 30 years	72	324	0.72 (0.57, 0.90)	
**Maternal work**				0.190
In employment	112	747	0.74 (0.59, 0.92)	
Without employment	110	201	0.85 (0.72, 1.02)	
**Maternal education**				0.132
Junior high school or below	97	183	0.77 (0.59, 1.01)	
Senior high shool or above	125	765	0.88 (0.74, 1.05)	
**Residence**				0.002
Urban	158	679	0.76 (0.63, 0.92)	
Rural	64	269	0.91 (0.75, 1.10)	
**Parity**				0.294
0	124	761	0.88 (0.74, 1.04)	
≥ 1	98	187	0.74 (0.58, 0.93)	
**Passive smoking**				0.378
No	144	860	0.83 (0.71, 0.97)	
Yes	78	88	0.72 (0.52, 1.00)	
**Anemia**				0.320
No	190	845	0.81 (0.70, 0.93)	
Yes	32	103	0.87 (0.72, 1.06)	
**Medication use**				0.138
No	134	660	0.83 (0.70, 0.98)	
Yes	88	288	0.78 (0.62, 0.98)	
**Folate/iron supplements use**				0.358
Yes	170	846	0.79 (0.67, 0.92)	
No	52	102	0.84 (0.65, 1.08)	
**Overall**	222	948	0.81 (0.71, 0.93)	

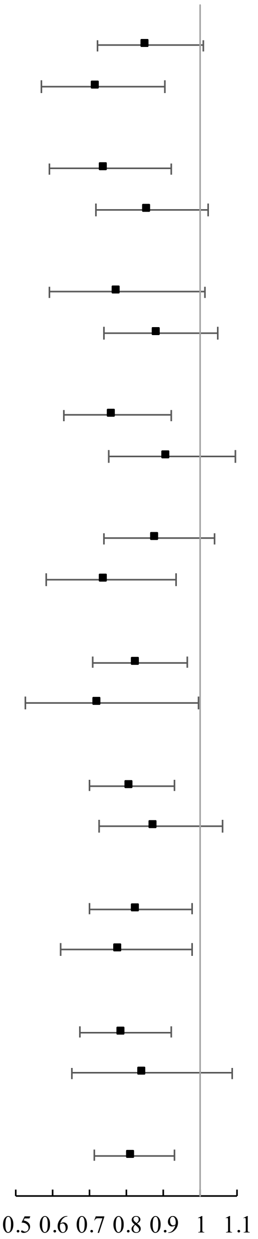

Figure S4. Subgroup analyses for the risk of ventricular septal defects associated with per 50 mg increase in total choline intake during pregnancy. Analyses were adjusted for total energy intake, maternal age, work, education, residence, parity, passive smoking, anemia, medication use, folate/iron supplements use, and dietary diversity score in pregnancy.

Subgroups	$N_{(cases)}$	$N_{(controls)}$	OR (95%CI)	P for interaction
**Maternal age**				0.389
<30 years	145	624	0.80 (0.68, 0.95)	
≥ 30 years	73	324	0.82 (0.67, 1.00)	
**Maternal work**				0.927
In employment	122	747	0.83 (0.68, 1.01)	
Without employment	96	201	0.78 (0.66, 0.93)	
**Maternal education**				0.587
Junior high school or below	70	183	0.84 (0.68, 1.03)	
Senior high shool or above	148	765	0.80 (0.68, 0.94)	
**Residence**				0.010
Urban	136	679	0.78 (0.64, 0.96)	
Rural	82	269	0.84 (0.70, 1.02)	
**Parity**				0.067
0	123	761	0.81 (0.68, 0.95)	
≥ 1	95	187	0.83 (0.67, 1.03)	
**Passive smoking**				0.080
No	153	860	0.77 (0.66, 0.89)	
Yes	65	88	0.84 (0.69, 1.03)	
**Anemia**				0.694
No	181	845	0.79 (0.69, 0.91)	
Yes	37	103	0.88 (0.72, 1.07)	
**Medication use**				0.721
No	126	660	0.79 (0.67, 0.93)	
Yes	92	288	0.83 (0.67, 1.02)	
**Folate/iron supplements use**				0.794
Yes	171	846	0.79 (0.68, 0.92)	
No	47	102	0.81 (0.62, 1.07)	
**Overall**	218	948	0.81 (0.71, 0.92)	

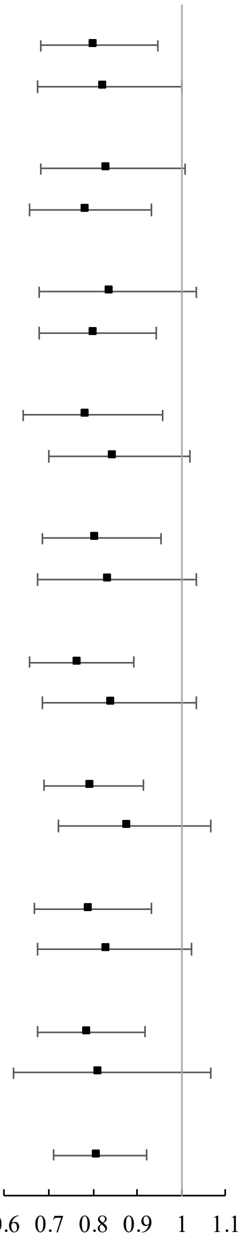

Figure S5. Subgroup analyses for the risk of atrial septal defects associated with per 50 mg increase in total choline intake during pregnancy. Analyses were adjusted for total energy intake, maternal age, work, education, residence, parity, passive smoking, anemia, medication use, folate/iron supplements use, and dietary diversity score in pregnancy.
